# Adhesion of Diarrheagenic *Escherichia coli* and Inhibition by Glycocompounds Engaged in the Mucosal Innate Immunity

**DOI:** 10.3390/biology2020810

**Published:** 2013-06-07

**Authors:** Alex L. Pereira, Loreny G. Giugliano

**Affiliations:** 1 College of Ceilândia, University of Brasília, Brasília-DF, 72220-900, Brazil; 2 Laboratory of Microbiology, Department of Cellular Biology, University of Brasília, Brasília-DF, 70910-900, Brazil; E-Mail: giuglian@unb.br

**Keywords:** diarrheagenic *Escherichia coli*, human milk, lactoferrin, secretory component, inhibition

## Abstract

*Escherichia coli* colonizes the human intestine shortly after birth, with most strains engaging in a commensal relationship. However, some *E. coli* strains have evolved toward acquiring genetic traits associated with virulence. Currently, five categories of enteroadherent *E. coli* strains are well-recognized, and are classified in regard to expressed adhesins and the strategy used during the colonization. The high morbidity associated with diarrhea has motivated investigations focusing on *E. coli* adhesins, as well on factors that inhibit bacterial adherence. Breastfeeding has proved to be the most effective strategy for preventing diarrhea in children. Aside from the immunoglobulin content, glycocompounds and oligosaccharides in breast milk play a critical role in the innate immunity against diarrheagenic *E. coli* strains. This review summarizes the colonization factors and virulence strategies exploited by diarrheagenic *E. coli* strains, addressing the inhibitory effects that oligosaccharides and glycocompounds, such as lactoferrin and free secretory components, exert on the adherence and virulence of these strains. This review thus provides an overview of experimental data indicating that human milk glycocompounds are responsible for the universal protective effect of breastfeeding against diarrheagenic *E. coli* pathotypes.

## 1. The Discovery of Diarrheagenic Categories of *Escherichia coli*

*Escherichia coli* is the predominant member of the *Proteobacteria* phylum and the dominant facultative anaerobe bacterium forming the gut microbiota [1]. The colonization of the gastrointestinal tract by commensal *E. coli* strains starts immediately after birth [2,3] with these commensal strains restricted to the distal ileum and colon [3]. *E. coli* is highly adapted to the gut environment and occupies a highly specific metabolic niche [1] in spite of the competition with hundreds of bacterial species that forms the crowded intestinal microbiota [2,4]. Most of the time, *E. coli* strains and their human host engage in a mutually beneficially relationship [5] and the bacterial strains remain restricted to the gut lumen and to the outer layer of the intestinal mucous [2,5]. However, there are some *E. coli* clones that have acquired genetic attributes conferring the ability to imbalance the healthy symbiosis accomplished by the microbiota and the mammalian host [5].

The recognition of diarrheagenic categories of *E. coli* began in the 1950’s when a series of papers showed that antigenically related strains were associated with infantile diarrhea [6,7]. As a result, specific O serogroups of *E. coli* have been associated with virulent strains since that time. However, O antigens themselves have no effective role in the virulence of the strains. In fact, *E. coli* serogroups only facilitate the identification of strains harboring virulence-associated genes [5]. In general, diarrheagenic *E. coli* strains express specific colonization factors that are not shared with commensal strains. In order to efficiently settle the gut mucosa, virulent strains exploit alternative host receptors avoiding the competition with the resident microbiota.

One of the first colonization factors specifically associated with diarrheagenic *E. coli* was described in enterotoxigenic *E. coli* (ETEC) strains [8]. These colonization factor antigens (CFA) allow ETEC strains to colonize the mucosal surfaces of the small bowel, the site of action of the ETEC enterotoxins heat-labile enterotoxin (LT) and heat-stable enterotoxin (ST). Indeed, in contrast to the type 1 pili, which were commonly harbored by commensal *E. coli* strains, fimbrial antigens CFA were characterized by mediating mannose-resistance hemagglutination (MRHA) [8,9]. 

**Figure 1:**
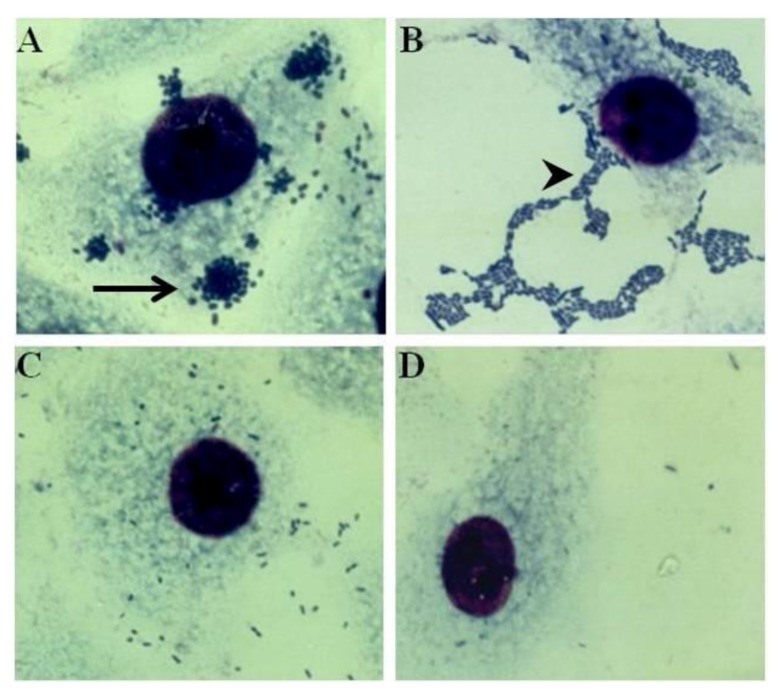
Distinctive adhesion patterns displayed by *E. coli* strains on HeLa cells when in the presence of D-mannose. (**A**) Localized adherence (LA) developed by a prototype EPEC strain. Note the formation of microcolonies (black arrow) on the host cell surface formed by tight clusters of bacterial cells. (**B**) Aggregative adherence (AA) formed by the prototype EAEC strain 042. AA phenotype is characterized by the autoagglutination of the bacterial cells into a conformation that resembles stacked bricks. The AA pattern develops on the edge of the host cell and extends forward on the abiotic surface (black arrowhead). (**C**) Diffusely adherent *E. coli* strain showing bacterial cells scattered on the surface of host cell as well as on the abiotic surface. (**D**) Non-adherent *E. coli* strain.

In the mid 80’s, the development of adhesion assays on epithelial cells revealed that diarrheagenic *E. coli* strains could use special adhesins that recognized non-mannosidic receptors in order to develop distinctive adhesion patterns [10]. It was shown that *E. coli* strains sharing classical EPEC serogroups O (O55, 086, O111, O119, O125, O128, and O142) developed a particular adherence pattern termed localized adherence (LA) when tested in the presence of D-mannose (Figure 1). In contrast, the mannose-resistant LA phenotype was not developed by ETEC strains harboring CFA fimbriae [10]. A subsequent study showed that the LA phenotype was mediated by an adhesion factor encoded by plasmids harboring a specific genetic sequence termed EAF (EPEC adherence factor) [11]. The identification of the plasmid-encoded EPEC adherence factor remained elusive until Girón *et al*. described its inducible nature in the presence of sheep blood or mammalian host cells [12]. The EPEC bundle-forming pili (BFP) were described as aggregated filaments capable of mounting a network of pili that mediated the formation of the localized adherence in *E. coli* strains belonging to classical EPEC serogroups (O55, O111, O127, O128, O142) [13]. The discovery of the LA pattern mediated by specific pili and developed by *E. coli* strains sharing classical EPEC serogroups was seminal not only for the EPEC research, but also for the discovery of another diarrheagenic category of *E. coli*.

Enteroaggregative *E. coli* (EAEC) strains were initially described as EAF-negative strains that were associated with infantile diarrhea and that displayed the distinctive adherence pattern named aggregative adherence (AA). AA phenotype was characterized by the autoagglutination of bacterial cells, described as stacked-brick configuration, that often occurred on the surface of host cells when in the presence of D-mannose (Figure 1) [5]. In a similar way as had occurred with EPEC, it was soon after reported that the AA pattern was mediated by specific fimbriae termed aggregative adherence fimbriae (AAF) that were encoded on high-molecular weight plasmids (pAA). Ultrastructural analysis of the AAF harbored by EAEC prototype strain 17-2 showed long and flexible bundle-forming fimbriae that connected bacterial cells together into aggregates and mediated hemagglutination of human erythrocytes [14].

By the time of the recognition of the AA phenotype, a third mannose-resistant adhesion pattern displayed by diarrhea-associated *E. coli* strains was defined as well as a new category of diarrheagenic strains. Diffusely adherent *E. coli* (DAEC) strains did not display the distinctive phenotypes LA or AA but, even so, they were able to both adhere to epithelial cells and agglutinate human erythrocytes in the presence of D-mannose (Figure 1). Thereafter, a genetic determinant for a new mannose-resistant adhesin (F1845) was characterized in a DAEC strain isolated from infantile diarrhea [15].

Studies of *E. coli* adhesion were essential not only for defining diarrheagenic categories but also for revealing the broad repertory of colonization factors used by pathogenic strains in order to colonize the gut mucosa. Currently, five enteroadherent *E. coli* pathotypes are well recognized. These diarrheagenic categories are characterized by virtue of their harboring specific colonization factors, expressing different adhesion patterns on cultured cells, and carrying out different strategies in order to colonize and persist on mucosal surfaces of the gut (Table 1) [1,5].

**Table 1:** Traits of the diarrheagenic pathotypes of enteroadherent *Escherichia coli*.

Pathotype	Histopathological feature	Major adhesion factors	Host cell receptor (adhesin)	Preferential gut site	Reference
Enterotoxigenic *E. coli* (ETEC)		Colonization factor antigens (CFA I, II and IV)	Sialoglycoprotein (CFA/I) Asialo ganglioside GM1 (CFA/II) Glycosphingolipid sulphatide (CFA/IV)	- Jejunal and duodenal mucosa	[16]
Enteropathogenic *E. coli* (EPEC)	- Localized adherence - Attachment-and-effacement lesion	Intimin α Bundle-forming pilus	β_1_-integrins (intimin α)	- Proximal and distal small intestine - Follicle associated epithelium of ileal Peyer’s patches	[17,18,19]
Enterohemorrhagic *E. coli* (EHEC)	- Attachment-and-effacement lesion	Intimin γ Long polar fimbriae (LPF)	β_1_-integrins (intimin γ) Fibronectin, laminin and collagen IV (LPF)	- Follicle associated epithelium of ileal Peyer’s patches	[17,18,19,20]
Enteroaggregative *E. coli* (EAEC)	- Aggregative adherence - Vesiculation of colonic microvilli	Aggregative adherence fimbriae (AAF-I to IV)	Glycoproteins (AAF-II): - thrombospondin-1—TSP1. - fibronectin - epidermal growth factor receptor—EGFR - endoplasmin—GRP-94/Gp96	- Ileal and colonic mucosa avoiding intestinal crypts	[21,22]
Diffusely adhering *E. coli* (DAEC)	- Diffuse adherence - Elongation of microvilli	Afa/Dr adhesins (AfaE alleles and F1845)	Glycosylphosphatidylinositol-anchored protein DAF (AfaE-I to IV and F1845) CEA-related cell adhesion molecule (AfaE-III and F1854)		[23,24,25]

**Abbreviations: DAF—**decay accelerating factor; **CEA**—carcinoembryonic antigen.

Besides the enteroadherent categories of *E. coli*, a pathotype of enteroinvasive strains are recognized as a distinctive group of diarrheagenic *E. coli*. Enteroinvasive *E. coli* (EIEC) is biochemically, genetically and pathogenically related to *Shigella* spp. EIEC and *Shigella* spp. bear remarkable phenotypic likeness, with a reduction in the number of substrates utilized in comparison to commensal and other pathotypes of *E. coli* strains. These similar phenotypes are attributed to the fact that these organisms spend much of their lifetime within eukaryotic cells experiencing a nutrient-rich and different environment from most *E. coli* strains. Recent phylogenetic studies have suggested that EIEC and *Shigella* ssp. form a single cluster of enteroinvasive pathogens. Moreover, EIEC strains are regarded as the precursors from which full-blown *Shigella* evolved [26]. As a result of this close evolutionary relatedness, EIEC shares the same pathogenesis model with *Shigella* ssp [1]. The illness caused by EIEC is characterized by the destruction of the colonic epithelium caused by the inflammatory response induced upon invasion of the mucosa by bacteria [27]. The early phase of EIEC pathogenesis comprises epithelial cell penetration, followed by lysis of the endocytic vacuole, intracellular multiplication, and directional movement through the cytoplasm toward adjacent epithelial cells. The escape from endocytic vacuole into the cytoplasm is followed by an actin-dependent motility process, which is mediated by nucleation of actin into a “tail” that extends from one pole of the bacterium, pushing bacteria toward adjacent epithelial cells [1,28,29]. The entire repertoire of genes required for entry into host cells is clustered in a 230-kb virulence-associated invasion plasmid (pINV) found in both *Shigella* and EIEC strains [1,30]. Due to its unique virulence mechanism among diarrheagenic *E. coli* strains, and the paucity of experimental data on inhibition of EIEC mediated by human milk glycocompounds, EIEC will not be further assessed in this review. 

## 2. Diarrheagenic Categories of *E. coli* Exploit Different Adhesins

Pathotypes of *E. coli* express specific adherence factors that allow the bacterial cells to explore eukaryotic cell receptors that commensal *E. coli* strains normally cannot do. These colonization factors are diverse in structure and they can be seen as hair-like polymeric structures that extend beyond the bacterial surface such as fimbriae, fibriallae and pili; or they can be present as non-polymeric afimbrial adhesins [1,5]. In addition, some adhesion factors can trigger signal transduction pathways what allows the bacteria to manipulate host cell functions during the pathogenesis [1,5].

### 2.1. Adhesins of Enterotoxigenic *Escherichia coli*

Enterotoxigenic *E. coli* (ETEC) is an important cause of diarrhea in children living in developing countries as well as a major cause of traveler`s diarrhea in adults that visit endemic areas. ETEC was the first diarrheagenic *E. coli* category for which virulence factors were described. It is now known that ETEC comprises a heterogeneous group of pathogens that have in common the ability to settle to the proximal small intestine (duodenal and jejunal mucosas), where they secrete the enterotoxins LT and/or ST [16,31]. ETEC adhesion factors have been proven to be important virulence attributes of this pathotype by allowing the colonization of gut sites that are susceptible to the produced enterotoxins. As such, the development of potential ETEC vaccines has been based mainly on the inhibition of the bacterial adhesion. The attachment of ETEC strains to host cells is mediated by a diverse group of colonization factors and, at least 21 different adhesion factors in human-isolated ETEC strains have been described. ETEC colonization factors are formed by antigenically different proteins (coli surface antigen (CS) that can be mounted on the cell surface into a variety of morphological types. Indeed, these colonization factors can be classified as fimbrial, fibrillar, bundle-forming and nonfimbrial adhesins (Table 2). Despite such diversity, around 50 to 75% of human-isolated ETEC strains express either the colonization factors CFA/I, CFA/II or CFA/IV [32,33].

**Table 2:** Characteristics of colonization factors commonly detected in human-isolated ETEC strains.

Biotype	Coli surface antigen	Colonization factor antigen	Morphology	Receptor	Reference
CFA/I	CfaB	CFA/I	Fimbrial	Sialoglycoprotein Nonacid glycosphingolipids	[34,35,36]
CFA/II	CS1	CS1	Fimbrial	Asialo ganglioside GM1	[37,38]
CFA/II	CS2	CS2	Fimbrial	Asialo ganglioside GM1	[31,38]
CFA/II	CS3	CS3	Fibrillar	Asialo ganglioside GM1	[37,38]
CFA/III	CS8	CFA/III	Fimbrial		[16]
CFA/IV	CS4	CS4	Fimbrial	Asialo ganglioside GM1	[16,38]
CFA/IV	CS5	CS5	Fimbrial (double-helical fibrils)		[16]
CFA/IV	CS6	CS6	Fibrillar, fimbrial or nonfimbrial	Fibronectin Glycosphingolipid sulphatide	[16,39,40]

**Abbreviations: CFA**—colonization factor antigen; **CS—**coli surface antigen; **GM1—**monosialotetrahexosylganglioside.

Among these, CFA/I fimbriae are the most studied colonization factors and are considered the archetypical adhesin in ETEC. CFA/I fimbriae are long (about 1 µm in length) and quite straight helical filaments formed by polymerization of the structural protein CfaB (pilin) displaying the adhesion protein (adhesin) CfaE on the tip of the filament [34]. Ultrastructural analysis showed that CFA/I is a polymorphic structure and can be visualized either as a helical filament with 7.4 nm in diameter or as a uncoiled and extended thin fimbria with 3 nm in diameter [34,41]. This plasticity could underlie the ability of the CFA/I fimbria to oppose the peristaltic motions and fluid movements of the intestinal tract [34].

CFA/I-expressing strains were phenotypically characterized by promoting mannose-resistance hemagglutination of human group A erythrocytes. Thereafter, it has shown that some ETEC isolates recovered from patients were incapable of agglutinating human erythrocytes; however they agglutinated bovine erythrocytes in the presence of mannose. It was suggested that these ETEC strains harbored a novel colonization factor named CFA/II. In ETEC strains, the term CFA may denote a heterogeneous array of surface antigens displaying adherence properties instead of referring to a particular colonization factor [16]. In fact, ETEC strains expressing CFA/II activity can display on the cell surface three colonization factors named CS1, CS2 or CS3 (Table 2). Among CFA/II-positive strains, the expression of CS3 and the co-expression of CS1 along with CS3 are the most frequent phenotypes detected globally in ETEC strains [32]. CS1 and CS3 subunits are immunologically distinct and are expressed as different morphological types. The CS1 appendage is assembled on the cell surface as a straight and rigid fimbria with 6 nm in diameter, while CS3 is displayed as thinner, wiry and flexible fimbria with 2 nm in diameter [37]. Morphological and phenotypical analyses showed that CS2 fimbriae are straight and rigid filaments with 6 nm in diameter that mediated the bacterial adherence to the jejunal mucosal cells, especially to the brush border [31].

Thought to be a single fimbria, CFA/IV was initially characterized for supporting mannose-resistance hemagglutination of human, bovine and guinea-pig cells in ETEC strains isolated from diarrheic humans [42]. Thereafter, it was shown that CFA/IV-positive strains harbored three distinct antigenic components termed CS4, CS5 and CS6 (Table 2) [16,42]. CS4 fimbriae were similar in morphology to the fimbriae formed by CfaB (CFA/I), CS1 and CS2, while CS5 fimbriae has a distinctive morphology and are displayed as a helical structure formed by two fine fibrils [16]. Variations in the morphological type assumed by CS6 have been detected; therefore, fimbrial and fibrillar structures and even the nonfimbrial nature of CS6 has been reported. In addition, recently it was shown that CS6 has a unique molecular structure among ETEC colonization factors formed by two major subunits (CssA and CssB) [39,43]. In CFA/IV strains, CS4 and CS5 accounted for the adhesion of the bacteria to the cultured human duodenal mucosa [16]. In a quite different way, the role of CS6 in the adherence of ETEC strains was demonstrated using culture of intestinal cell lineages [44,45]. Moreover, it was showed that CS6 is capable of binding the glycosphingolipid sulfatide that is the major acid glycosphingolipid detected in enterocytes from human small gut [40].

### 2.2. Adhesins in A/E Lesion-Inducing *E. coli* Pathotypes

Attachment-and-effacement (A/E) lesion is a distinctive gut mucosa histopathology that was initially associated with infection by enteropathogenic *E. coli* (EPEC), but now known to be caused by enterohemorrhagic *E. coli* (EHEC) as well. This complex physiopathological process is initiated with the intimate adhesion of bacterium cell to the intestinal epithelial cell followed by an intense reshuffle of the host cytoskeleton highlighted by the accumulation of polymerized actin directly beneath the adherent bacterium [1,5]. In EPEC strains, the physiopathological process toward the A/E lesion is dependent on a series of adhesion factors that initially mediate the bacterial autoaggregation during the formation of bacterial microcolonies (LA phenotype) and, thereafter, the intimate contact of the bacterium with the host cell.

Bundle-forming pilus (BFP) was the first EPEC colonization factor that was described mediating the formation of microcolonies (LA phenotype) on the host cell [12]. BFP is displayed as long filaments (15 to 20 µm long) that aggregate laterally to form bundles reaching 50 to 500 nm in diameter [12]. BFP is essential in the early stages of the bacterial adhesion as well as in the development of the microcolonies. Besides supporting bacterial autoaggregation, BFP mediate the initial attachment of bacterial clusters to the host cells, showing a species-specific interaction with human-derived intestinal cell lines [46,47,48]. After the initial formation of the microcolonies on the host cell, the expression of BFP is diminished, bacterial clusters become looser and larger, and EPEC cells begin to spread on the host cell [46]. In addition, it is shown that host cellular extracts stimulate the release of BFP from the adherent bacterial cluster favoring the bacterial dispersal [46]. The microcolony dispersion is also important during the EPEC pathogenesis since it allows bacteria to colonize additional mucosal sites [49]. A volunteer study proved that the dynamics involving the BFP expression is important for the EPEC pathogenesis [49]. Mutant strains defective in producing BFP as well as BFP hyperpiliated EPEC strains have their ability to cause diarrhea attenuated [49].

During the pathogenesis by EPEC strains, bacteria can contact the host cells employing the EspA filament, the component of type III secretion system (TTSS) responsible for the translocation of effector proteins into the host cell. Since this filament is thought to mediate a physical connection between planktonic bacteria and host cell, EspA has been considered a colonization factor in EPEC strains [48]. Using BFP mutant strains, it was shown that EspA mediates the initial adhesion of EPEC to the brush border of intestinal cells, although the formation of microcolonies was not observed [48]. When these mutant strains were complemented with a copy of the BFP gene, the ability to form microcolonies was restored showing that EspA have a trivial role as prime adherence factor during the development of LA phenotype [48].

The intimate contact of EPEC strains with the host cell is mediated by the adhesin intimin, encoded by *eae* genes. Intimin recognizes a bacterial receptor (Tir) that is translocated into the host cell via TTSS EspA filament and, thereafter, is inserted into the host cell membrane [1,5]. Although it is not necessary for the expression of LA phenotype, the intimin-mediated adhesion is essential for the development of A/E lesion, and BFP-mutant strains possessing a functional TTSS are still capable of inducing A/E lesion in the host cell [48]. In EHEC strains, which do not express BFP, intimin is the single adherence factor proved to play a pivotal role in the formation of A/E lesions [17].

Although A/E lesion is a common hallmark in EPEC and EHEC strains, these pathotypes express antigenically distinct variants of intimin and colonize different intestinal sites (Table 1) [17,18]. At least five subtypes of intimin have been well characterized and are designated α, β, γ, δ and ε [50]. Among these subtypes, intimin α is specifically associated with the clone 1 of EPEC strains while intimin γ are frequently found in EHEC O157 and its related strains (O55:H7) [17,18,50]. Studies have shown that the receptor binding function of different types of intimin is supported by the *C*-terminal 280 amino acids (Int280), which display a three dimensional topology homologous to the C-type lectins, a protein family responsible for the recognition of carbohydrate moieties on the eukaryotic cell surface [51,52]. Besides Tir, intimin binds directly to receptors expressed by epithelial cell lines (HeLa and HEp-2 cells) and this binding is dependent on a disulphide bridge at the carboxy terminus of the Int280. It has been proposed that β_1_ integrins would be the potential cell host receptors for intimin, given that Int208 can bind to β_1_ integrins purified from human mononuclear cells and that some animal intestinal cells also express β_1_ integrins [19]. Moreover, in intestinal cell line T84, the infection of EPEC strains causes the redistribution of β_1_ integrins from the basolateral to the apical cell membrane where they are bound by intimin [53]. Variations in the subtypes of intimins support the tissue specificity carried out by EPEC and EHEC strains during the colonization of the intestinal mucosa [17,18]. EPEC strains expressing intimin α are able to colonize different parts of intestine, including the proximal and distal small intestine and the follicle associated epithelium (FAE) of ileal Peyer’s patches as well as sections of the large intestine (colon) [17,18]. Conversely, EHEC strains harboring intimin γ have a distinctive tropism for the follicle associated epithelium (FAE) of ileal Peyer’s patches (Table 1) [17,18].

Besides intimin, two colonization factors closely related to the long polar fimbriae of *Salmonella enterica* serovar Typhimurium have been described in EHEC strains [54,55]. Long polar fimbriae (Lpf 1 and 2) mediate the adhesion of EHEC strains to cultured cells (HEp-2 and T84) and also to human intestinal explants. Results obtained from *in vitro* organ culture have shown that Lpf mediates the EHEC adhesion to the FAE of ileal Peyer’s patches [56]. Additionally, mutations in *lpf* loci impair the intestinal colonization by EHEC strains in animal models [57]. Lpf1 binds to the extracellular matrix proteins fibronectin, laminin and collagen IV, which are commonly found in the intestinal mucosa, and therefore improves the EHEC adherence to intestinal cultured cells [20].

### 2.3. Adhesins in Enteroaggregative *E. coli*

EAEC strains were defined as *E. coli* that display the aggregative adherence (AA) on epithelial cell lines (Hep-2 and HeLa) and that do not secret the enterotoxins LT or ST. This definition encompasses a heterogeneous group of strains formed by both pathogenic and non-pathogenic clones [1,5]. In prototype strains, the AA phenotype is supported by the expression of the aggregative adherence fimbriae (AAF), which are related to the superfamily of Dr adhesins on the basis of genetic architecture displayed by the AAF operons (chaperone-usher-cryptic gene-subunit) and due to the presence of conserved residues located on the *N*-terminus of the adhesin [58]. In prototype EAEC strains, AAF operons are carried by pAA plasmids that also harbor the major transcriptional activator of virulence termed AggR. The term “typical EAEC” has been coined to refer to a subgroup of supposed pathogenic isolates of EAEC that harbor the AggR regulon [1].

Nowadays, there are four well-known AAF adhesins expressed by prototype strains. These fimbriae display different structures when expressed on the bacterial cell surface. Aggregative adherence fimbriae I (AAF-I) are expressed forming long and flexible filaments with a diameter of 2 nm that usually form bundles [14]. AAF-II are thicker than AAF-I (5 nm in diameter) and are expressed as rigid structures commonly found as loose bundle of filaments [58]. Contrary to AAF-I and II, AAF-III are displayed as long and flexible filaments (3–5 nm in diameter) observed without forming bundles [59]. In prototype strains, the expression of AAF’s are sufficient *per se* to support the AA phenotype on the epithelial cells (Figure 1). In addition, AAF variants mediate mannose-resistant hemagglutination of a broad range of species including erythrocytes from human (type A erythrocytes), bovine, guinea pig, rabbit (only AAF-I and II) and sheep (only AAF-I and III) [14,58,59,60]. AAF-IV, previously known as HdaA, was first reported to mediate the AA phenotype and hemagglutination displayed by EAEC strains isolated from Danish children [60]. Genetic sequence analysis performed with Dr adhesins showed that AAF-IV makes up a phylogenetic cluster along with the adhesins Afa-8 and M-agglutinin, while the AAF variants I to III are grouped into a distinct phylogenetic branch [60]. Despite the genetic divergence, AAF-IV is sufficient for triggering innate inflammatory response and neutrophil transmigration in intestinal xenograft model as do the prototype fimbriae AAF-I to III [61].

Recently, it was reported that a set of glycoproteins derived from the human intestinal cell line INT-407 are recognized by AAF-II. These host receptors for AAF-II are identified as fibronectin, epidermal growth factor receptor (EGFR), the extracellular matrix protein TSP1 (thrombospondin-1) and GRP-94 (endoplasmin) (Table 1) [62]. It was suggested that the adherence of EAEC strains to host cells involve multiple receptors and that a co-operative action among receptors is required for bacterial adhesion. Infection assays performed in the presence of antibodies against each identified receptor showed that the reduction in bacterial adhesion was 76% when TSP1 was targeted [62]. When fibronectin, EGFR or GRP-94 were blocked, the reduction in the bacterial adhesion mediated by AAF-II was about 30% [62]. Besides recognizing cellular receptors, AAFs are able to support unspecific adhesion of EAEC strains to abiotic surfaces such as polystyrene and glass coverslips [14,58].

In addition to allelic variants of AAF that are known today, multiples colonization factors have been described to mediate the AA phenotype in wild-type EAEC strains, including fimbrial and non-fimbrial adhesins. Furthermore, epidemiological studies have shown that the prototype AAF adhesins are present in only a minority of the wild type EAEC strains [1,60,63,64,65]. An afimbrial adhesin, named Ap58 (aggregative protein 58), has been identified in EAEC strains of serotype O111:H12 that were isolated from Brazilian children with diarrhea [66]. In these strains, which were negative for AAF alleles, Ap58 mediated the bacterial adherence to the epithelial cells (HEp-2) and supported the agglutination of bovine erythrocytes, both in the presence of D-mannose [66].

In spite of that heterogeneity, infection models have proved that EAEC strains share the ability to mount biofilms forming a thick layer of autoaggregating bacteria that adhere to the ileal and, predominantly, to the colonic mucosa [21,22]. On the colonic mucosa, EAEC strains exhibit a tissue tropism, colonizing primarily the luminal mucosa but not the areas around the intestinal crypts (Table 1) [22]. While colonizing the ileal mucosa, EAEC strains induce a high level production of intestinal mucus, and bacterial cells are found forming aggregates in association with the mucus layer that overlies the cellular brush border [21].

Given the genetic heterogeneity presented by EAEC strains, the expression of biofilms has been considered the consensual virulence factor among isolates of this pathotype. Biofilm formation is a multi-stepped and complex process that may involve several factors. In addition, the discovery that factors not devoted to adhesion are also important in EAEC biofilm has highlighted its multifactorial nature. An AAF-independent mechanism for biofilm formation was described in an atypical EAEC strain (C1096), which has been implicated as the causative agent of a neonatal diarrhea outbreak [67]. EAEC strain C1096 exhibits biofilm formation as well as adherence to epithelial cells mediated by a plasmid-encoded type IV pilus (PilS), a multifunctional factor involved in numerous phenotypes in Gram-negative pathogens including conjugation [67]. Moreover, we have shown that typical EAEC strains recovered from diarrhea form biofilms supported by the expression of putative F pili [68]. Long (2 µm in length) and flexible pili were seen to mediate the bacteria-to-bacteria interaction and the attachment of bacteria to abiotic surface during the biofilm formation by EAEC strains [68].

### 2.4. Adhesins in Diffusely Adhering *E. coli*

Even today, diffusely adherent *Escherichia coli* (DAEC) is considered a heterogeneous group of isolates of which supposed diarrheagenic *E. coli* strains would be included [23,69,70]. DAEC strains are defined as the *E. coli* isolate that displays a scattered pattern of adherence (diffuse adherence—DA) to the host epithelial cells (HeLa or HEp-2) (Figure 1) that is mediated by colonization factors belonging to the Afa/Dr family of adhesins [1]. Afa/Dr operons have been detected in strains displaying a variety of genetic backgrounds including both pathogenic and commensal strains [23,70]. In addition, the Afa/Dr gene *afaE*, which encodes the adhesin, is a highly variable gene causing the expression of antigenically distinct adhesins, with five described *afaE* alleles (1 through 3, 5 and 8) [23]. Indeterminate *afaE* genes have also been reported in DAEC strains [70,71,72]. Meanwhile different collections of DAEC strains were appraised, including strains recovered from children and adults; Mansan-Almeida et al. found 20% of the Afa/Dr-positive strains carried indeterminate *afaE* genes [70].

Afa/Dr adhesins are mainly expressed as afimbrial adhesins with the exception of the AfaE-1 and F1845 that show fibrillar structures [23,73]. Afa/Dr adhesins interact with three well-recognized host cell receptors. Dr adhesin binds to type-IV collagen and, unlike other Afa/Dr adhesins, supports bacterial adhesion that is sensitive to chloranphenicol [73]. Some Afa/Dr adhesins (AfaE-III, Dr and F1845) bind to the cellular glycoprotein named carcinoembryonic antigen (CAE) and members of a subfamily of related molecules (CEACAM—CEA-related cell adhesion molecules) mediating the bacterial adhesion (Table 1) [23]. Despite its name, CEA is a normal epithelial molecule that is highly expressed by goblet cells of the large and small intestines, by columnar cells in the large intestine and by microfold cells (M cells) of the colonic mucosa [74]. In relation to CEACAM, CEACAM1 molecule is equally expressed by all epithelial cells of the small and large intestine besides the M cells [74]. As common with other GPI-anchored proteins, CEA molecules can trigger cellular responses by signal transduction. Therefore, it has been shown that CEA is mobilized and gathered underneath DAEC strains while bacterial cells adhere to the brush border of human polarized intestinal cells (Caco-2 cells) [75]. The best-characterized host cell receptor exploited by Afa/Dr DAEC strains is the decay-accelerating factor (DAF), a glycosylphosphatidylinositol-anchored protein, which normally protects cells from damage by the complement system (Table 1). With the exception of AfaE adhesins expressed by DAEC strains from animals species (AfaE-7 and 8), all Afa/Dr adhesins recognized DAF as a receptor [23,73]. DAF is extensively found, not only in epithelial membranes of gastrointestinal mucosa, but also, in epithelial surfaces of genitourinary tract [73]. Studies employing intestinal cell lines (Caco-2 and INT407) have shown that the fimbrial adhesin F1845 expressed by DAEC strains binds to DAF mediating the bacterial adherence to the host cell [24,25]. In the Caco-2 cell line, the binding of F1845 to DAF induces injury to microvilli (MV) characterized by the development of long cellular extensions, which enfold the adherent bacteria [25]. Moreover, the MV injuries result from a disassembly of the actin network in the apical and basal cell domains [25]. In the INT407 cell line, the actin rearrangement is supported by signal transduction involving phosphatidylinositol 3-kinase (PI-3 kinase) [24].

## 3. Impairing Virulence by Targeting *E. coli* Adhesins

Diarrhea diseases remain an important cause of morbidity worldwide and, in developing countries, constitute an important public health concern because of the high mortality affecting children. During the 1990s, it was estimated that around 1.4 billion cases of diarrhea occurred every year among children up to five years of age [76]. Nowadays, it is estimated that 1.3 million diarrhea-associated deaths occurred annually in this age group, primarily in economically underprivileged areas [77]. In developing areas, categories of diarrheagenic *E. coli* are the most common bacterial agent causing diarrhea in children, accounting for about 40% of all cases [76]. In this scenario, research focusing on the inhibition of *E. coli* adherence has gained great appeal. In virtue of the plethora of colonization factors displayed by *E. coli* pathotypes, the development of anti-adhesion vaccines intending to block the adhesin-receptor interactions has proved to be a hard task. Particularly with ETEC strains, which are the most common cause of diarrhea in developing areas, the existence of multiples colonization factors (CF), along with the fact that several of these CF are co-expressed by the same bacterium, has made the development of a universal and effective anti-adhesion vaccine for ETEC a complex issue [78,79]. On the other hand, breast-feeding is one of the most cost-effective strategies known for the prevention of morbidity and mortality caused by infectious disease affecting children, including respiratory-tract infection and diarrheal diseases [77,80,81].

The protective effect of human milk is mediated by the combinatory action of acquired and innate defense factors. The immunoglobulin fraction (mainly secretory immunoglobulin A—sIgA) is responsible for the acquired immunity that is dependent on previous contact with the etiologic agent. In the mid 1990s, Giugliano and co-workers proposed that glycocompounds could contribute to the innate defense inhibiting the adherence of enteropathogens [82]. The authors reported that lactoferrin (Lf) and free secretory component (fSC), both isolated from human milk, inhibited the adhesion to erythrocytes (hemagglutination) mediated by a CFA1-positive ETEC strain [82]. Today, it is well recognized that human milk glycocompounds as well as unconjugated forms of oligossacharides take part in the innate response against diarrheagenic *E. coli* strains isolated from humans (Table 3) [83,84,85,86,87,88,89].

**Table 3:** Effect of human milk glycocompounds and free oligosaccharides on the diarrheagenic categories of *E. coli*.

Innate immune factor	Target *E. coli* pathotype	Inhibited adhesion/colonization processes	Interactions with adhesins	Reference
Lactoferrin	ETEC	- Inhibition of CFA-I-mediated hemagglutination - Inhibition of the ETEC adherence to epithelial cells - Inhibition of the gut colonization	- Binds to the CFA-I but do not to CFA-II	[82,85,90]
	EPEC	- Inhibition of EPEC adhesion to epithelial cells - Inhibition of the A/E lesion - Inhibition of EPEC-induced hemolysis	- Binds to EspA and EspB promoting proteolytic degradation	[86,87,88,91]
	EHEC	- Inhibition of the gut colonization and of the spreading toward the kidney (mouse infection model)		[92]
	EAEC	- Inhibition of EAEC adhesion to epithelial cells - Inhibition of biofilm formation	- Promotes release of AAF-II and its degradation	[89,93]
	DAEC	- Inhibition of adhesion to epithelial cells displayed by DAEC harboring F1845		[89]
Secretory component	ETEC	- Inhibition of CFA-I-mediated hemagglutination	- Binds to the CFA-I and CFA-II	[82,85]
	EPEC	- Inhibition of EPEC adhesion to epithelial cells - Inhibition of the A/E lesion	- Binds to intimin α	[86,94]
Sialyl (acid)-oligosaccharide	ETEC	- Inhibition of CFA-I- and CFA-II-mediated hemagglutination		[88]
	EPEC	- Inhibition of EPEC adhesion to Caco-2 cells		[84]
Neutral oligosaccharide	EPEC	- Inhibition of EPEC adhesion to Caco-2 cells		[84]

**Abbreviations: A/E lesion**—attachment-and-effacement lesion; **F1845—**prototype Afa/Dr fimbria; **CFA—**colonization factor antigen of ETEC.

### 3.1. Inhibition of Diarrheagenic *E. coli* by Lactoferrin

Lf is an 80-kDa glycosylated protein (ca. 700 amino acids) with high homology among species and found in high concentration in human colostrum (ca. 7 g/L); it is the second most abundant protein in milk, after the caseins. Lf, which binds to two ferric ions (Fe^3+^), is recognized as a member of transferrin family [95,96]. Additionally, Lf is a multifunctional protein that is involved in many physiological processes including iron absorption in the gut, modulation of inflammatory processes and protection against microbial infections [95]. The sequestration of iron by Lf deprives the microorganisms of this essential nutrient and produces a bacteriostatic effect. In addition to the antimicrobial effect, many studies have shown that Lf, when used at sub-inhibitory concentrations, can bind to and block several colonization factors displayed by diarrheagenic types of *E. coli* (Table 3).

Lf inhibits the adhesion of ETEC strains to the human epithelial cells as well as the colonization of the intestinal tract in animal models [90]. The capacity of Lf to specifically bind to ETEC colonization factors was showed using enriched fimbrial extracts in immunolabeling assays followed by electron microscopy analyses. It has showed that Lf binds to CFA-I extracts, however Lf does not bind to CFA-II fimbriae (Table 3) [85].

Concerning EPEC strains, Lf inhibits the bacterial adherence to human epithelial cells (HeLa and HEp-2) [86,91] and actin polymerization, and therefore prevents the development of the A/E lesion [91]. It has been shown that Lf interacts with the structural subunits of the type III secretion system (TTSS) promoting the degradation of both EspA (the filament-forming subunit) and EspB (the pore-forming subunit) (Table 3) [87]. In addition, Lf reduces the gene expression of EspA about 50% during EPEC strains infection of epithelial cells [91]. Studies of EHEC strain O157:H7 in animal infection models (BALB/c mice), showed that Lf has a protective effect against gut colonization and the spreading toward kidneys [92]. Therefore, animals treated with Lf had a reduction in the mortality rate of 50%, and kidney injuries were also eased [92]. 

In the early 2000s, epidemiological studies involving children and adult travelers showed that EAEC-associated diarrhea was followed by a significant increase in the fecal excretion of Lf [97,98]. At that time, the increase in fecal Lf was merely seen as a marker of intestinal inflammation. Thereafter, it was reported that Lf inhibited the adhesion to epithelial cells displayed by EAEC strains recovered from diarrhea [89,93]. Biofilm formation supported by the EAEC strain 042, which express AAF-II, is also inhibited by Lf when tested in non-lethal concentrations [93]. Lf induces the release of AAF-II from the bacterial surface and promotes proteolytic degradation of the released fimbriae (Table 3) [93]. In relation to DAEC strains, a single paper has reported that Lf impairs the adhesion of a DAEC strain harboring fimbriae F1845 to epithelial cells [89].

### 3.2. Inhibition of *E. coli* Adhesion by Free Secretory Component (FSC)

The secretory component (SC) is an integral part of the secretory IgA (sIgA) that supports the translocation of IgA from mucosal epithelial cells into external fluids [99]. Bound to the IgA, SC anchors the immunoglobulin molecule to the Ig receptor located in the basolateral membrane, and after transcytosis has occurred, it is cleaved away from the receptor releasing sIgA [100]. Free secretory component (fSC) is found in human milk as a consequence of the continuous process of transport that occurs even in the absence of IgA [99,100]. Thereafter, fSC is abundantly found in human milk as an uncomplexed 80-kDa glycoprotein, consisting of a single polypeptide chain with a large amount of carbohydrate residues (around 20%) that branches from seven *N*-glycosylation sites on the polypeptide [94,100].

The first clues on the anti-adhesion proprieties of fSC were brought to light by Giugliano`s paper which showed that human milk-purified fSC (at a concentration of 0.06 mg/mL) inhibited the hemagglutination mediated by CFA-I-positive ETEC strains [82]. A subsequent report endorsed those findings [85]. It was showed that human milk-purified fCS specifically binds to fimbrial extracts of CFA-I and CFA-II (CS-1 and CS-3) (Table 3) [85].

In regard to EPEC strains, it has been showed that human milk-purified fSC reduces the adhesion of EPEC strains to HeLa cells by 32% when tested at a concentration of 105 µg/mL [86]. On the other hand, the immunoglobulin fraction, containing majority sIgA and residues of casein, inhibited the adhesion of EPEC by only 12% when tested at a concentration of 320 µg/mL. Purified casein, tested at a concentration of 300 µg/mL, did not inhibit the adhesion of EPEC [86]. In addition, purified human recombinant SC (SCrec), expressed in mammals cells, inhibits the adhesion of EPEC to HeLa cells as well as the development of A/E lesion (Table 3) [94]. The binding of SCrec to intimin α has been confirmed and showed to be dependent on the oligosaccharide moieties on the SC molecule [94]. Using differentially deglycosylated SC, it was showed that galactose and sialic acid residues were necessary for the interaction with intimin, but fucose residues were not [94].

### 3.3. Inhibition of *E. coli* Adhesion by Human Milk Oligosaccharides

Quantitatively, oligosaccharades are, all together, the third component of human milk, besides lactose and lipids. They are synthesized by specific glycosyltransferase that promote the sequential addition of fucose, galactose, acetylglycosamines or sialic acid to molecules of lactose [101]. The huge potential for structural isomers suggests that there may be thousands of potential human milk oligosaccharide structures, what increases the probability that some of them have active biological roles [84,101]. Some papers have showed that human milk oligosaccharides can inhibit the adherence ability of diarrheagenic *E. coli* strains (Table 3) [83,84,88,101].

It was reported that the fraction from human milk containing sialylated oligosaccharide (sialyl-oligosccharide) inhibits the hemagglutination mediated by CFA-I-expressing ETEC strains and CFA-II as well (Table 3) [88]. Experimental data showed that sialic acid residues displayed by oligosaccharides had a pivotal role in the inhibitory effect. Oligosaccharide fractions that were treated with sialidases (neuraminidase) had their inhibitory effect on the ETEC adherence drastically reduced. Individually, different sialylated oligosaccharides can display specific inhibitory effect against a single CFA variant. The oligosaccharide 6'-sialyllactose (6'SL) specifically blocks the erythrocyte adherence mediated by CFA-I-expressing strains, but does not block the adherence mediated by CFA-II. Conversely, the oligosaccharide 3'-sialyllactose (3'SL) inhibits only the adherence supported by CFA-II. In addition, 3'-sialyl-3-fucosyllactose (3'S3FL), sialyllacto-*N*-tetraose a and disialyllacto-*N*-tetraose (LSTa) showed a broader inhibition effect blocking the hemagglutination mediated by both CFA-I and CFA-II fimbriae [88].

In 1991, Cravioto and co-workers reported that oligosaccharide-enriched fractions extracted from human milk showed an inhibitory effect on adhesion of EPEC strains to HEp-2 cell; however, adherence of EAEC and DAEC strains were not inhibited. Using partially purified fractions, it was proposed that EPEC strains attach to host cells through the preferential recognition of carbohydrates displaying fucosylated residues [102]. In relation to the effect of human milk oligosaccharides on the EPEC adherence, it was showed that sialyl (acidic)-oligosaccharides and neutral oligosaccharides reduce the EPEC adherence to Caco-2 cells (Table 3) [84]. Considering the sialyl-oligosaccharide pooled fraction, the reduction on the EPEC adherence was 36%. When isolate sialyl-oligosaccharide was tested, the glycans 3'SL and 6'SL inhibited the adhesion of EPEC by 28% and 16% respectively [84]. Neutral oligosaccharides also displayed inhibitory effect on EPEC adherence with the glycan 3-fucosyllactose (3-FL) inhibiting the adhesion by 30% [84]. It is important to note that single tested monosaccharides (glucose, galactose, sialic acid, *N*-acetylglucosamine and fucose), which human milk oligosaccharides were composed of, did not inhibit the adhesion of EPEC strain to the Caco-2 cells [84].

## 4. Conclusions

By the late 1950s, a series of papers claimed that breastfed children experienced a lower frequency of diarrhea episodes than artificially-fed children [103]. These epidemiological findings supported the assumption that human milk acted beneficially inducing host resistance to intestinal infections. At that time, despite the fact that the protective factors in human milk had not been clearly identified, there was a strong belief that the protection involved, exclusively, anti-microbial factors such as the immunoglobulin content (mostly, the content of secretory IgA) and, even, the presence of IgA-synthesizing lymphocytes in the human milk [103]. A seminal study conducted with Mayan Indian women, reported that 70% of the milk samples displayed appreciable antibody titers (greater than 1:32) against antigen O displayed by enteropathogens, however these titers declined shortly after colostrums feeding period [103,104]. In addition, antibodies against the EPEC serogroup O111 were detected either in the lowest titers or it could be, sporadically, not detected even in the colostrum samples. Even though the protection imputed to the immunoglobulin fraction works on the dependence of mother’s immunological experience, an enlightening paper endorsed the innate protective role of breastfeeding against diarrhea [105,106]. The paper showed that unprocessed human milk had been successfully employed as a single measure for controlling a diarrhea outbreak caused by *E. coli* O111 which had affected newborns for 6 months [105,106].

In the last three decades, the idea that human milk glycocompounds, lactoferrin and secretory component, play a role as innate defense factors against diarrheagenic *E. coli* categories has been verified by a series of independent studies. Besides its bacteriostatic effect, it has been documented that lactoferrin interacts with a broad range of adhesion factors expressed by ETEC, EPEC, EAEC and DAEC strains and, therefore, inhibits the colonization of host cells by these diarrheagenic *E. coli* pathotypes. Regarding the secretory component (SC), in addition to its function in transporting sIgA, experimental data has shown that SC also binds to colonization factors expressed by ETEC and EPEC. Moreover, free oligosaccharides that compose human milk also inhibit the adhesion of ETEC and EPEC strains. Given the huge number of colonization factors, as well as the antigenic variation displayed by diarrheagenic *E. coli* strains, the existence of these innate defense factors inhibiting bacterial adhesion confirms the universal protective effect that historically has been attributed to human milk against infantile diarrhea. In addition, the importance of lactoferrin and secretory component in protecting against diarrhea extends to other species of enteropathogens as has been shown for *Shigella* and *Salmonella* species [107,108].

## References

[1] Kaper J.B., Nataro J.P., Mobley H.L. (2004). Pathogenic *Escherichia coli*. Nat. Rev. Microbiol..

[2] Sekirov I., Russell S.L., Antunes L.C., Finlay B.B. (2010). Gut microbiota in health and disease. Physiol. Rev..

[3] Fanaro S., Chierici R., Guerrini P., Vigi V. (2003). Intestinal microflora in early infancy: Composition and development. Acta Paediatr. Suppl..

[4] Qin J., Li R., Raes J., Arumugam M., Burgdorf K.S., Manichanh C., Nielsen T., Pons N., Levenez F., Yamada T. (2010). A human gut microbial gene catalogue established by metagenomic sequencing. Nature.

[5] Nataro J.P., Kaper J.B. (1998). Diarrheagenic *Escherichia coli*. Clin. Microbiol. Rev..

[6] Orskov F. (1951). On the occurrence of *E. coli* belonging to O-group 26 in cases of infantile diarrhoea and white scours. Acta Pathol. Microbiol. Scand..

[7] Ewing W.H., Tanner K.E., Tatum H.W. (1955). A new serotype of *Escherichia coli* associated with infantile diarrhea. Public Health Rep..

[8] Evans D.G., Silver R.P., Evans D.J., Chase D.G., Gorbach S.L. (1975). Plasmid-controlled colonization factor associated with virulence in *Esherichia coli* enterotoxigenic for humans. Infect. Immun..

[9] Evans D.G., Evans D.J. (1978). New surface-associated heat-labile colonization factor antigen (CFA/II) produced by enterotoxigenic *Esherichia coli* of serogroups O6 and O8. Infect. Immun..

[10] Scaletsky I.C., Silva M.L., Trabulsi L.R. (1984). Distinctive patterns of adherence of enteropathogenic *Esherichia coli* to HeLa cells. Infect. Immun..

[11] Nataro J.P., Scaletsky I.C., Kaper J.B., Levine M.M., Trabulsi L.R. (1985). Plasmid-mediated factors conferring diffuse and localized adherence of enteropathogenic *Esherichia coli*. Infect. Immun..

[12] Giron J.A., Ho A.S., Schoolnik G.K. (1991). An inducible bundle-forming pilus of enteropathogenic *Esherichia coli*. Science.

[13] Giron J.A., Ho A.S., Schoolnik G.K. (1993). Characterization of fimbriae produced by enteropathogenic *Esherichia coli*. J. Bacteriol..

[14] Nataro J.P., Deng Y., Maneval D.R., German A.L., Martin W.C., Levine M.M. (1992). Aggregative adherence fimbriae I of enteroaggregative *Esherichia coli* mediate adherence to HEp-2 cells and hemagglutination of human erythrocytes. Infect. Immun..

[15] Bilge S.S., Clausen C.R., Lau W., Moseley S.L. (1989). Molecular characterization of a fimbrial adhesin, F1845, mediating diffuse adherence of diarrhea-associated *Esherichia coli* to HEp-2 cells. J. Bacteriol..

[16] Knutton S., McConnell M.M., Rowe B., McNeish A.S. (1989). Adhesion and ultrastructural properties of human enterotoxigenic *Esherichia coli* producing colonization factor antigens III and IV. Infect. Immun..

[17] Fitzhenry R.J., Pickard D.J., Hartland E.L., Reece S., Dougan G., Phillips A.D., Frankel G. (2002). Intimin type influences the site of human intestinal mucosal colonisation by enterohaemorrhagic *Esherichia coli* O157:H7. Gut.

[18] Phillips A.D., Frankel G. (2000). Intimin-mediated tissue specificity in enteropathogenic *Esherichia coli* interaction with human intestinal organ cultures. J. Infect. Dis..

[19] Frankel G., Lider O., Hershkoviz R., Mould A.P., Kachalsky S.G., Candy D.C., Cahalon L., Humphries M.J., Dougan G. (1996). The cell-binding domain of intimin from enteropathogenic *Esherichia coli* binds to beta1 integrins. J. Biol. Chem..

[20] Farfan M.J., Cantero L., Vidal R., Botkin D.J., Torres A.G. (2011). Long polar fimbriae of enterohemorrhagic *Esherichia coli* O157:H7 bind to extracellular matrix proteins. Infect. Immun..

[21] Hicks S., Candy D.C., Phillips A.D. (1996). Adhesion of enteroaggregative *Esherichia coli* to pediatric intestinal mucosa *in vitro*. Infect. Immun..

[22] Knutton S., Shaw R.K., Bhan M.K., Smith H.R., McConnell M.M., Cheasty T., Williams P.H., Baldwin T.J. (1992). Ability of enteroaggregative *Esherichia coli* strains to adhere *in vitro* to human intestinal mucosa. Infect. Immun..

[23] Le B.C., Servin A.L. (2006). Diffusely adherent *Esherichia coli* strains expressing Afa/Dr adhesins (Afa/Dr DAEC): hitherto unrecognized pathogens. FEMS Microbiol. Lett..

[24] Peiffer I., Servin A.L., Bernet-Camard M.F. (1998). Piracy of decay-accelerating factor (CD55) signal transduction by the diffusely adhering strain *Esherichia coli* C1845 promotes cytoskeletal F-actin rearrangements in cultured human intestinal INT407 cells. Infect. Immun..

[25] Bernet-Camard M.F., Coconnier M.H., Hudault S., Servin A.L. (1996). Pathogenicity of the diffusely adhering strain *Esherichia coli* C1845: F1845 adhesin-decay accelerating factor interaction, brush border microvillus injury, and actin disassembly in cultured human intestinal epithelial cells. Infect. Immun..

[26] Lan R., Alles M.C., Donohoe K., Martinez M.B., Reeves P.R. (2004). Molecular evolutionary relationships of enteroinvasive *Esherichia coli* and Shigella spp. Infect. Immun..

[27] Parsot C. (2005). Shigella spp. and enteroinvasive *Esherichia coli* pathogenicity factors. FEMS Microbiol. Lett..

[28] Sansonetti P. (2002). Host-pathogen interactions: The seduction of molecular cross talk. Gut.

[29] Sansonetti P.J. (2001). Rupture, invasion and inflammatory destruction of the intestinal barrier by Shigella, making sense of prokaryote-eukaryote cross-talks. FEMS Microbiol. Rev..

[30] Sansonetti P.J., D’Hauteville H., Ecobichon C., Pourcel C. (1983). Molecular comparison of virulence plasmids in Shigella and enteroinvasive *Esherichia coli*. Ann. Microbiol. (Paris).

[31] Honda T., Cakir N., Arita M., Miwatani T. (1989). Purification and characterization of the CS2 pili of colonization factor antigen II produced by human enterotoxigenic *Esherichia coli*. Microbiol. Immunol..

[32] Isidean S.D., Riddle M.S., Savarino S.J., Porter C.K. (2011). A systematic review of ETEC epidemiology focusing on colonization factor and toxin expression. Vaccine.

[33] Wolf M.K. (1997). Occurrence, distribution, and associations of O and H serogroups, colonization factor antigens, and toxins of enterotoxigenic *Esherichia coli*. Clin. Microbiol. Rev..

[34] Mu X.Q., Savarino S.J., Bullitt E. (2008). The three-dimensional structure of CFA/I adhesion pili: Traveler’s diarrhea bacteria hang on by a spring. J. Mol. Biol..

[35] Pieroni P., Worobec E.A., Paranchych W., Armstrong G.D. (1988). Identification of a human erythrocyte receptor for colonization factor antigen I pili expressed by H10407 enterotoxigenic *Esherichia coli*. Infect. Immun..

[36] Jansson L., Tobias J., Lebens M., Svennerholm A.M., Teneberg S. (2006). The major subunit, CfaB, of colonization factor antigen i from enterotoxigenic *Esherichia coli* is a glycosphingolipid binding protein. Infect. Immun..

[37] Levine M.M., Ristaino P., Sack R.B., Kaper J.B., Orskov F., Orskov I. (1983). Colonization factor antigens I and II and type 1 somatic pili in enterotoxigenic *Esherichia coli*: Relation to enterotoxin type. Infect. Immun..

[38] Oro H.S., Kolsto A.B., Wenneras C., Svennerholm A.M. (1990). Identification of asialo GM1 as a binding structure for *Esherichia coli* colonization factor antigens. FEMS Microbiol. Lett..

[39] Roy S.P., Rahman M.M., Yu X.D., Tuittila M., Knight S.D., Zavialov A.V. (2012). Crystal structure of enterotoxigenic *Esherichia coli* colonization factor CS6 reveals a novel type of functional assembly. Mol. Microbiol..

[40] Jansson L., Tobias J., Jarefjall C., Lebens M., Svennerholm A.M., Teneberg S. (2009). Sulfatide recognition by colonization factor antigen CS6 from enterotoxigenic *Esherichia coli*. PLoS One.

[41] Li Y.F., Poole S., Nishio K., Jang K., Rasulova F., McVeigh A., Savarino S.J., Xia D., Bullitt E. (2009). Structure of CFA/I fimbriae from enterotoxigenic *Esherichia coli*. Proc. Natl. Acad. Sci. USA.

[42] Thomas L.V., McConnell M.M., Rowe B., Field A.M. (1985). The possession of three novel coli surface antigens by enterotoxigenic *Esherichia coli* strains positive for the putative colonization factor PCF8775. J. Gen. Microbiol..

[43] Wolf M.K., de Haan L.A., Cassels F.J., Willshaw G.A., Warren R., Boedeker E.C., Gaastra W. (1997). The CS6 colonization factor of human enterotoxigenic *Esherichia coli* contains two heterologous major subunits. FEMS Microbiol. Lett..

[44] Ghosal A., Bhowmick R., Banerjee R., Ganguly S., Yamasaki S., Ramamurthy T., Hamabata T., Chatterjee N.S. (2009). Characterization and studies of the cellular interaction of native colonization factor CS6 purified from a clinical isolate of enterotoxigenic *Esherichia coli*. Infect. Immun..

[45] Tobias J., Lebens M., Kallgard S., Nicklasson M., Svennerholm A.M. (2008). Role of different genes in the CS6 operon for surface expression of Enterotoxigenic *Esherichia coli* colonization factor CS6. Vaccine.

[46] Tobe T., Sasakawa C. (2001). Role of bundle-forming pilus of enteropathogenic *Esherichia coli* in host cell adherence and in microcolony development. Cell Microbiol..

[47] Tobe T., Sasakawa C. (2002). Species-specific cell adhesion of enteropathogenic *Esherichia coli* is mediated by type IV bundle-forming pili. Cell Microbiol..

[48] Cleary J., Lai L.C., Shaw R.K., Straatman-Iwanowska A., Donnenberg M.S., Frankel G., Knutton S. (2004). Enteropathogenic *Esherichia coli* (EPEC) adhesion to intestinal epithelial cells: Role of bundle-forming pili (BFP), EspA filaments and intimin. Microbiology.

[49] Bieber D., Ramer S.W., Wu C.Y., Murray W.J., Tobe T., Fernandez R., Schoolnik G.K. (1998). Type IV pili, transient bacterial aggregates, and virulence of enteropathogenic *Esherichia coli*. Science.

[50] Oswald E., Schmidt H., Morabito S., Karch H., Marches O., Caprioli A. (2000). Typing of intimin genes in human and animal enterohemorrhagic and enteropathogenic *Esherichia coli*: Characterization of a new intimin variant. Infect. Immun..

[51] Kelly G., Prasannan S., Daniell S., Fleming K., Frankel G., Dougan G., Connerton I., Matthews S. (1999). Structure of the cell-adhesion fragment of intimin from enteropathogenic *Esherichia coli*. Nat. Struct. Biol..

[52] Frankel G., Candy D.C., Fabiani E., du-Bobie J., Gil S., Novakova M., Phillips A.D., Dougan G. (1995). Molecular characterization of a carboxy-terminal eukaryotic-cell-binding domain of intimin from enteropathogenic *Esherichia coli*. Infect. Immun..

[53] Muza-Moons M.M., Koutsouris A., Hecht G. (2003). Disruption of cell polarity by enteropathogenic *Esherichia coli* enables basolateral membrane proteins to migrate apically and to potentiate physiological consequences. Infect. Immun..

[54] Torres A.G., Giron J.A., Perna N.T., Burland V., Blattner F.R., velino-Flores F., Kaper J.B. (2002). Identification and characterization of lpfABCC'DE, a fimbrial operon of enterohemorrhagic *Esherichia coli* O157:H7. Infect. Immun..

[55] Torres A.G., Kanack K.J., Tutt C.B., Popov V., Kaper J.B. (2004). Characterization of the second long polar (LP) fimbriae of *Esherichia coli* O157:H7 and distribution of LP fimbriae in other pathogenic *E. coli* strains. FEMS Microbiol. Lett..

[56] Fitzhenry R., Dahan S., Torres A.G., Chong Y., Heuschkel R., Murch S.H., Thomson M., Kaper J.B., Frankel G., Phillips A.D. (2006). Long polar fimbriae and tissue tropism in *Esherichia coli* O157:H7. Microb. Infect..

[57] Jordan D.M., Cornick N., Torres A.G., an-Nystrom E.A., Kaper J.B., Moon H.W. (2004). Long polar fimbriae contribute to colonization by *Esherichia coli* O157:H7 *in vivo*. Infect. Immun..

[58] Czeczulin J.R., Balepur S., Hicks S., Phillips A., Hall R., Kothary M.H., Navarro-Garcia F., Nataro J.P. (1997). Aggregative adherence fimbria II, a second fimbrial antigen mediating aggregative adherence in enteroaggregative *Esherichia coli*. Infect. Immun..

[59] Bernier C., Gounon P., Le B.C. (2002). Identification of an aggregative adhesion fimbria (AAF) type III-encoding operon in enteroaggregative *Esherichia coli* as a sensitive probe for detecting the AAF-encoding operon family. Infect. Immun..

[60] Boisen N., Struve C., Scheutz F., Krogfelt K.A., Nataro J.P. (2008). New adhesin of enteroaggregative *Esherichia coli* related to the Afa/Dr/AAF family. Infect. Immun..

[61] Boll E.J., Struve C., Sander A., Demma Z., Nataro J.P., McCormick B.A., Krogfelt K.A. (2012). The fimbriae of enteroaggregative *Esherichia coli* induce epithelial inflammation *in vitro* and in a human intestinal xenograft model. J. Infect. Dis..

[62] Konar M., Sachin O., Priya A., Ghosh S. (2012). Identification of key proteins of cultured human intestinal cells involved in interaction with enteroaggregative *Esherichia coli*. FEMS Immunol. Med. Microbiol..

[63] Piva I.C., Pereira A.L., Ferraz L.R., Silva R.S., Vieira A.C., Blanco J.E., Blanco M., Blanco J., Giugliano L.G. (2003). Virulence markers of enteroaggregative *Esherichia coli* isolated from children and adults with diarrhea in Brasilia, Brazil. J. Clin. Microbiol..

[64] Pereira A.L., Ferraz L.R., Silva R.S., Giugliano L.G. (2007). Enteroaggregative *Esherichia coli* virulence markers: Positive association with distinct clinical characteristics and segregation into 3 enteropathogenic *E. coli* serogroups. J. Infect. Dis..

[65] Boisen N., Scheutz F., Rasko D.A., Redman J.C., Persson S., Simon J., Kotloff K.L., Levine M.M., Sow S., Tamboura B. (2012). Genomic characterization of enteroaggregative *Esherichia coli* from children in Mali. J. Infect. Dis..

[66] Monteiro-Neto V., Bando S.Y., Moreira-Filho C.A., Giron J.A. (2003). Characterization of an outer membrane protein associated with haemagglutination and adhesive properties of enteroaggregative *Esherichia coli* O111:H12. Cell Microbiol..

[67] Dudley E.G., Abe C., Ghigo J.M., Latour-Lambert P., Hormazabal J.C., Nataro J.P. (2006). An IncI1 plasmid contributes to the adherence of the atypical enteroaggregative *Esherichia coli* strain C1096 to cultured cells and abiotic surfaces. Infect. Immun..

[68] Pereira A.L., Silva T.N., Gomes A.C., Araujo A.C., Giugliano L.G. (2010). Diarrhea-associated biofilm formed by enteroaggregative *Esherichia coli* and aggregative Citrobacter freundii: A consortium mediated by putative F pili. BMC Microbiol..

[69] Tacket C.O., Moseley S.L., Kay B., Losonsky G., Levine M.M. (1990). Challenge studies in volunteers using *Esherichia coli* strains with diffuse adherence to HEp-2 cells. J. Infect. Dis..

[70] Mansan-Almeida R., Pereira A.L., Giugliano L.G. (2013). Diffusely adherent *Esherichia coli* strains isolated from children and adults constitute two different populations. BMC Microbiol..

[71] Arikawa K., Meraz I.M., Nishikawa Y., Ogasawara J., Hase A. (2005). Interleukin-8 secretion by epithelial cells infected with diffusely adherent *Esherichia coli* possessing Afa adhesin-coding genes. Microbiol. Immunol..

[72] Fujihara S., Arikawa K., Aota T., Tanaka H., Nakamura H., Wada T., Hase A., Nishikawa Y. (2009). Prevalence and properties of diarrheagenic *Esherichia coli* among healthy individuals in Osaka City, Japan. Jpn. J. Infect. Dis..

[73] Servin A.L. (2005). Pathogenesis of Afa/Dr diffusely adhering *Esherichia coli*. Clin. Microbiol. Rev..

[74] Baranov V., Hammarstrom S. (2004). Carcinoembryonic antigen (CEA) and CEA-related cell adhesion molecule 1 (CEACAM1), apically expressed on human colonic M cells, are potential receptors for microbial adhesion. Histochem. Cell Biol..

[75] Guignot J., Peiffer I., Bernet-Camard M.F., Lublin D.M., Carnoy C., Moseley S.L., Servin A.L. (2000). Recruitment of CD55 and CD66e brush border-associated glycosylphosphatidylinositol-anchored proteins by members of the Afa/Dr diffusely adhering family of *Esherichia coli* that infect the human polarized intestinal Caco-2/TC7 cells. Infect. Immun..

[76] O'Ryan M., Prado V., Pickering L.K. (2005). A millennium update on pediatric diarrheal illness in the developing world. Semin. Pediatr. Infect. Dis..

[77] Lamberti L.M., Fischer Walker C.L., Noiman A., Victora C., Black R.E. (2011). Breastfeeding and the risk for diarrhea morbidity and mortality. BMC Public Health.

[78] Svennerholm A.M., Tobias J. (2008). Vaccines against enterotoxigenic *Esherichia coli*. Exp. Rev. Vaccines..

[79] Tobias J., Svennerholm A.M. (2012). Strategies to overexpress enterotoxigenic *Esherichia coli* (ETEC) colonization factors for the construction of oral whole-cell inactivated ETEC vaccine candidates. Appl. Microbiol. Biotechnol..

[80] Work Group on Breastfeeding (1997). Breastfeeding and the use of human milk—American Academy of Pediatrics. Pediatrics.

[81] Gartner L.M., Morton J., Lawrence R.A., Naylor A.J., O’Hare D., Schanler R.J., Eidelman A.I. (2005). Breastfeeding and the use of human milk. Pediatrics.

[82] Giugliano L.G., Ribeiro S.T., Vainstein M.H., Ulhoa C.J. (1995). Free secretory component and lactoferrin of human milk inhibit the adhesion of enterotoxigenic *Esherichia coli*. J. Med. Microbiol..

[83] Morrow A.L., Ruiz-Palacios G.M., Jiang X., Newburg D.S. (2005). Human-milk glycans that inhibit pathogen binding protect breast-feeding infants against infectious diarrhea. J. Nutr..

[84] Coppa G.V., Zampini L., Galeazzi T., Facinelli B., Ferrante L., Capretti R., Orazio G. (2006). Human milk oligosaccharides inhibit the adhesion to Caco-2 cells of diarrheal pathogens: *Esherichia coli*, Vibrio cholerae, and Salmonella fyris. Pediatr. Res..

[85] Oliveira I.R., de Araujo A.N., Bao S.N., Giugliano L.G. (2001). Binding of lactoferrin and free secretory component to enterotoxigenic *Esherichia coli*. FEMS Microbiol. Lett..

[86] De Araujo A.N., Giugliano L.G. (2001). Lactoferrin and free secretory component of human milk inhibit the adhesion of enteropathogenic *Esherichia coli* to HeLa cells. BMC Microbiol..

[87] Ochoa T.J., Noguera-Obenza M., Ebel F., Guzman C.A., Gomez H.F., Cleary T.G. (2003). Lactoferrin impairs type III secretory system function in enteropathogenic *Esherichia coli*. Infect. Immun..

[88] Martin-Sosa S., Martin M.J., Hueso P. (2002). The sialylated fraction of milk oligosaccharides is partially responsible for binding to enterotoxigenic and uropathogenic *Esherichia coli* human strains. J. Nutr..

[89] Nascimento de A.A., Giugliano L.G. (2000). Human milk fractions inhibit the adherence of diffusely adherent *Esherichia coli* (DAEC) and enteroaggregative *E. coli* (EAEC) to HeLa cells. FEMS Microbiol. Lett..

[90] Kawasaki Y., Tazume S., Shimizu K., Matsuzawa H., Dosako S., Isoda H., Tsukiji M., Fujimura R., Muranaka Y., Isihida H. (2000). Inhibitory effects of bovine lactoferrin on the adherence of enterotoxigenic *Esherichia coli* to host cells. Biosci. Biotechnol. Biochem..

[91] Flores-Villasenor H., Canizalez-Roman A., de la G.M., Nazmi K., Bolscher J.G., Leon-Sicairos N. (2012). Lactoferrin and lactoferrin chimera inhibit damage caused by enteropathogenic *Esherichia coli* in HEp-2 cells. Biochimie.

[92] Flores-Villasenor H., Canizalez-Roman A., Velazquez-Roman J., Nazmi K., Bolscher J.G., Leon-Sicairos N. (2012). Protective effects of lactoferrin chimera and bovine lactoferrin in a mouse model of enterohaemorrhagic *Esherichia coli* O157:H7 infection. Biochem. Cell Biol..

[93] Ochoa T.J., Brown E.L., Guion C.E., Chen J.Z., McMahon R.J., Cleary T.G. (2006). Effect of lactoferrin on enteroaggregative *E. coli* (EAEC). Biochem. Cell Biol..

[94] Perrier C., Sprenger N., Corthesy B. (2006). Glycans on secretory component participate in innate protection against mucosal pathogens. J. Biol. Chem..

[95] Gonzalez-Chavez S.A., Arevalo-Gallegos S., Rascon-Cruz Q. (2009). Lactoferrin: Structure, function and applications. Int. J. Antimicrob. Agents.

[96] Vogel H.J. (2012). Lactoferrin, a bird's eye view. Biochem. Cell Biol..

[97] Steiner T.S., Lima A.A., Nataro J.P., Guerrant R.L. (1998). Enteroaggregative *Esherichia coli* produce intestinal inflammation and growth impairment and cause interleukin-8 release from intestinal epithelial cells. J. Infect. Dis..

[98] Greenberg D.E., Jiang Z.D., Steffen R., Verenker M.P., DuPont H.L. (2002). Markers of inflammation in bacterial diarrhea among travelers, with a focus on enteroaggregative *Esherichia coli* pathogenicity. J. Infect. Dis..

[99] Snoeck V., Peters I.R., Cox E. (2006). The IgA system: A comparison of structure and function in different species. Vet. Res..

[100] Phalipon A., Corthesy B. (2003). Novel functions of the polymeric Ig receptor: Well beyond transport of immunoglobulins. Trends Immunol..

[101] Newburg D.S. (2000). Oligosaccharides in human milk and bacterial colonization. J. Pediatr. Gastroenterol. Nutr..

[102] Cravioto A., Tello A., Villafan H., Ruiz J., del V.S., Neeser J.R. (1991). Inhibition of localized adhesion of enteropathogenic *Esherichia coli* to HEp-2 cells by immunoglobulin and oligosaccharide fractions of human colostrum and breast milk. J. Infect. Dis..

[103] Mata L.J., Wyatt R.G. (1971). The uniqueness of human milk. Host resistance to infection. Am. J. Clin. Nutr..

[104] Caceres A., Mata L.J. (1970). Indirect hemagglutination for the study of antibodies to enterobacteriaceae. Rev. Latinoam. Microbiol..

[105] Hanson L.A., Winberg J. (1972). Breast milk and defence against infection in the newborn. Arch. Dis. Child.

[106] Jelliffe D.B. (1971). Active anti-infective properties of human milk. Lancet.

[107] Bessler H.C., de Oliveira I.R., Giugliano L.G. (2006). Human milk glycoproteins inhibit the adherence of *Salmonella typhimurium* to HeLa cells. Microbiol. Immunol..

[108] Willer E.M., Lima R.L., Giugliano L.G. (2004). *In vitro* adhesion and invasion inhibition of Shigella dysenteriae, Shigella flexneri and Shigella sonnei clinical strains by human milk proteins. BMC Microbiol..

